# Ex-post analysis of landraces sympatric to a commercial variety in the center of origin of the potato failed to detect gene flow

**DOI:** 10.1007/s11248-014-9854-4

**Published:** 2014-11-29

**Authors:** M. Ghislain, J. D. Montenegro, H. Juarez, M. del Rosario Herrera

**Affiliations:** 1 International Potato Center, P.O. Box 1558, Lima 12, Peru; 2 Present Address: International Potato Center, P.O. Box 25171, Nairobi, 00603 Kenya; 3 Present Address: School of Agriculture and Food Science, University of Queensland, Brisbane, QLD 4072 Australia

**Keywords:** Transgenic, Gene flow, Center of origin, Potato, Biodiversity

## Abstract

The possible introduction of genetically modified potato in the Andean region raises concerns about the unintentional introduction of transgenes into the native potato germplasm because it is perceived to convey negative impacts on biodiversity. We investigated this question by an ex-post analysis of existing landraces resulting from natural hybridization between an unknown landrace and the fertile commercial variety ‘Yungay’. This variety can be regarded as exotic because it was bred in part from the southern Chilean germplasm of *Solanum tuberosum* Group Chilotanum. We sampled the landrace germplasm of 1,771 leaf samples comprising more than 400 different landraces from three regions where ‘Yungay’ and landraces have coexisted for 15–25 years in the Peruvian Andes. Simple sequence repeat (SSR) markers were used to identify putative hybrids based on allele sharing with those of ‘Yungay’. The exclusion procedure was iterative, starting with the SSR markers with highest discriminating capacity based on allele frequency of the variety ‘Yungay’ in our large database of 688 landraces by 24 SSR makers. With only 12 of the 24 SSR markers assayed, all of the samples could be rejected as possible hybrids with ‘Yungay’ as a parent. This result demonstrates that the unintentional introduction of a transgene, not under farmers’ selection, from a widely grown transgenic variety over a long period of time is unlikely to happen at a detectable scale. Our finding reinforces the prominent role of farmers in the selection and maintenance of landraces which, unlike hybrids, have specific characteristics that farmers appreciate.

## Introduction

Crops with genetic modification (GM) using recombinant DNA technology (i.e., transgenic crops) have now been on the market for almost two decades. Technology adopters and producers have benefited substantially. The environment stands to gain, too, through reduced negative impacts of pesticides, pests, and tilling practices. New methods of genetic modification using genes from the crop’s own gene pool or using gene-editing tools are the next generation of GM crops. They should be less controversial, as the products could, in theory, be similar to those derived from conventional breeding or mutagenesis. Despite their economic, social, and other benefits, GM crops remain largely controversial due primarily to the perception that they invariably harm the environment.

Environmental risk assessment of transgenic crops considers the impact of the transgenic crop and the new trait on non-target organisms as well as on biodiversity. Gene flow and its impact on the crops’ germplasm that farmers maintain in the center of origin and (often) diversity are still the technology’s most controversial feature—and the one that elicits the strongest animosity. Gene flow of transgenes into crop’s germplasm is repeatedly cited as a serious concern despite the absence of substantiated evidence of occurrence and level of damage.

The case of potato in the Andes is as emblematic of this controversy as maize is in Mexico. The possible cultivation of transgenic varieties of potato has raised the concern of the persistence of the transgenes into the native gene pool through sexual hybridization between transgenic varieties and local cultivars as well as wild species. Farmers may recognize and appreciate particular GM trait into a hybrid plant which might eventually result in new landraces. Whether this presence is harmful on the crop’s biodiversity or not has been poorly investigated. Entrenched personal antagonism by many toward GM technology and a public naïve about its underlying science have slowed the push for broader field research.

Globally, potato is the fourth most important food crop and is cultivated in more than 150 countries around the world. It is grown rainfed or irrigated in different environments with night temperatures of 8°–15 °C and from sea level to 4,000 masl. Potato is susceptible to a large array of pests and diseases, which are mainly controlled through chemical treatment and crop management practices. Host-plant resistance plays a much smaller role in controlling pathogens than it does for other cereals crops. What is more, new varieties are developed more slowly for a number of reasons: (1) potato is clonally propagated by farmers, (2) the processing industry demands uniform varieties to ensure stability and consistency of tuber qualities, and (3) potato’s complex genetics slow the progress of conventional breeding. However, the ability to transfer foreign genes into widely accepted varieties has long been exploited to add traits such as resistance to pests and diseases (e.g., Colorado potato beetle, leafroll, X and Y viruses), and modified starch. This first generation of released GM, or transgenic potato varieties, is now entering a next generation with many more engineered traits of interest to farmers and consumers in developing countries. These varieties show resistance to late blight, tuber moth, viruses, nematodes, and weevils, and are low in acrylamide and high in beta-carotene (Barrell et al. 2013).

The potato *Solanum tuberosum* L. belongs to the section Petota, subsection Potatoe series Tuberosa. It was domesticated sometime between 6,000 (oldest fossilized remains) and 10,000 years ago when agriculture started, somewhere in South America where tuber-bearing wild Solanum species existed. The most plausible origin is in the Andes of southern Peru from wild species in the *Solanum brevicaule* complex (Spooner et al. 2005). Farmers have been the main factor in this process of domestication and enrichment of their cultivated potatoes with germplasm from wild species (Quiros et al. 1992).

The domestication and diversification of the cultigen from diploid to pentaploids result from introgression, interspecific hybridization, auto- and allopolyploidy, and sexual compatibility with many wild species. Yet the sequence and timeframe for how this all happened are still unknown. Primitive diploid potatoes were found to hybridize abundantly with wild species (Rabinowitz et al. 1990). Crossability in potato depends on several factors, but compatibility is wide among cultivated germplasm between diploid and tetraploid landraces which are also self-compatible (Andersson and de Vicente 2009). Interspecific compatibility has been often associated with post-zygotic conditions at the endosperm level (Jackson and Hanneman 1999). The frequent occurrence of unreduced gametes (2n) in diploid potatoes facilitates gene flow across ploidies. Production of berries is frequent and end up buried in the field, where 150–250 million seeds per hectare stay viable from 7 to 20 years (Lawson 1983). One of the so-called bitter diploid potatoes, *S. ajanhuiri*, would have been created in a region in the department of La Paz in Bolivia (Johns and Keen 1986). Landraces, also referred to as native potatoes, are cultivated potatoes resulting from indigenous breeding. These belong to several cultivar groups and ploidy levels, formerly described as species and subspecies, whose molecular characterization revealed their hybrid nature (Spooner and Hetterscheid 2006; Rodríguez et al. 2010).

There is no accurate inventory of all landraces in the Andes; defining what makes a cultivated potato a landrace is difficult. Indigenous names of landraces for the same potato change from village to village, different landraces may share the same names, and cultivated area changes from year to year. The International Potato Center (CIP), however, has a representative sample of approximately 5,000 accessions collected from all over the Andes. This collection is certainly only a fraction of the global native potato diversity (de Haan et al. 2010).

The cultivation of GM, or transgenic, varieties in the center of origin and diversity of the potato has long been strongly opposed. This resistance is based upon cultural values as well as scientific arguments that gene flow would likely occur under certain biological and physical conditions and thus alter the natural gene pool and diminish biodiversity. But only gene flow has been investigated, not the fate of foreign genes and their impact on biodiversity. In a first well-designed study in Peru, three of the six wild species occurring near cultivated potato fields were confirmed to produce 3–4 % of hybrid seeds naturally according to amplified fragment length polymorphisms fingerprinting when sympatric to commercial potato varieties (Celis et al. 2004). In a follow-up study by Scurrah et al. (2008), other wild species were included. Again, hybrid seeds were produced naturally from the commercial potato variety ‘Yungay’ at frequencies of 2.9, 2.4, and 14.4 % with *S. acaule*, *S. bukasovii*, and *S. sparsipilum*, respectively. However, none of the seedlings emerging from field-planted berries survived the biotic and abiotic stresses. The authors demonstrated that farmers were interested in keeping some of the hybrids produced and thereby could include them in their native potato germplasm.

The introduction of commercial varieties in the Andean agro-ecosystem may in fact have already changed the original or native gene pool of landraces grown in the same fields or nearby. Wild species that are sexually compatible may have changed as well. Therefore, unintentional introgression of exotic genes could have already taken place. The central question here is, are Andean farmers, custodians of the native potato germplasm, unknowingly actually preserving hybrids between landraces and commercial varieties? To improve the understanding of the issues concerning gene flow in the potato’s center of origin and diversity, we have searched evidence of past events of gene flow into potato landraces in regions where these coexisted with commercial varieties. Ghislain et al. (2009a) have genotyped a large set of landraces and varieties using SSR markers and generated a large database. A core set of 24 SSR markers were characterized for their technical reproducibility, single locus, genome coverage, and information content—in particular, absence of null allele (Ghislain et al. 2009b). We used this marker technology and an exclusion procedure similar to the one Ellstrand (1984) used to assess multiple paternity of wild radish.

## Materials and methods

### Selection of the commercial variety

The choice of the commercial potato variety was dictated by four criteria: (1) the variety represents a typical improved variety deriving from the *Chilotanum* Group of *S. tuberosum* that is suitable for the urban and processing market; (2) both male and female are fertile; (3) the variety was cultivated for many years in several regions of the country; and (4) the variety is cultivated close to landraces.

### Potato cultivar group distribution data

We used all records from CIP’s genebank that included cultivar groups and passport data of potato. Passport data include a description of the location of origin and geographic coordinates. The presence of coordinate data allowed the analysis of richness of potato cultivar groups. Data were assigned to grid cells using a circular neighborhood (Bonham-Carter 1994) with a radius of 50 km through the use of the DIVA-GIS software (Hijmans et al. 2001).

### Actual distribution of the commercial potato variety ‘Yungay’

Potato area statistics were obtained from the Ministry of Agriculture in 2007 and computed at district level (third administrative boundary). Survey data where the improved variety ‘Yungay’ grows were obtained from Maldonado et al. (2008) and computed at department level (first administrative boundary). Actual distribution of the improved variety ‘Yungay’ is the combination of these two layers. We assumed that ‘Yungay’ area was evenly distributed over the potato production zones.

### Sampling sites

A purposive, non-probabilistic sampling model was selected due to the nature of our objective and the geography of the locations selected. Expert advice from potato agronomists, university professors, and extension agents guided us in the choice of specific locations. Hence, our sampling method is dependent on information available about coexistence between commercial potato ‘Yungay’ and landraces within their respective area of distribution. Farmers were surveyed and GIS coordinates taken. In the case of Cuzco, we missed the cropping season at the time of collection activities; however, a sample was made available to us from a previous collection activity from farmers growing landraces in our targeted locations. The Cuzco sampling represents 100 landraces.

### SSR assay

Leaves in good physiological conditions were harvested directly in the field, put on ice, and transported by bus while still on ice until next-day extraction at the laboratory. Total DNA was extracted using the CTAB method modified to avoid the use of liquid nitrogen, and then used for SSR amplifications following previously published protocol (Herrera and Ghislain 2013).

### Screening for potential hybrid

The SSR profile of the selected commercial variety was obtained from CIP’s database consisting of 688 potato accessions by 24 SSR markers (Ghislain et al. 2009b). The 23 SSR markers from the Potato Genetic Identity Kit (PGI-Kit) were first used to screen the samples (Ghislain et al. 2009a). Additional SSR markers were identified from this database, considering those markers with many alleles of low frequency. The exclusion procedure of Ellstrand (1984) was applied to this paternity testing in potato. Because the hybrid is an F1, only landraces that share at least one allele with the commercial variety ‘Yungay’ were kept for further screening. We did not use the more stringent criteria of two alleles sharing because many alleles have the same size for which differences in band intensity could, in theory, be used for allele dosage but in practice may not always be reliable. A second reason is to account for a mutation event, which is always possible, albeit rare. Because SSR marker scoring was dominant due to technical difficulty of accurately determining allele dosage, a selection criterion for accepting hypothesis of possible hybrid was at least one allele shared with the commercial potato variety. Screening continued until all samples were eliminated as possible hybrids.

## Results

### Sampling sites

**Fig. 1:**
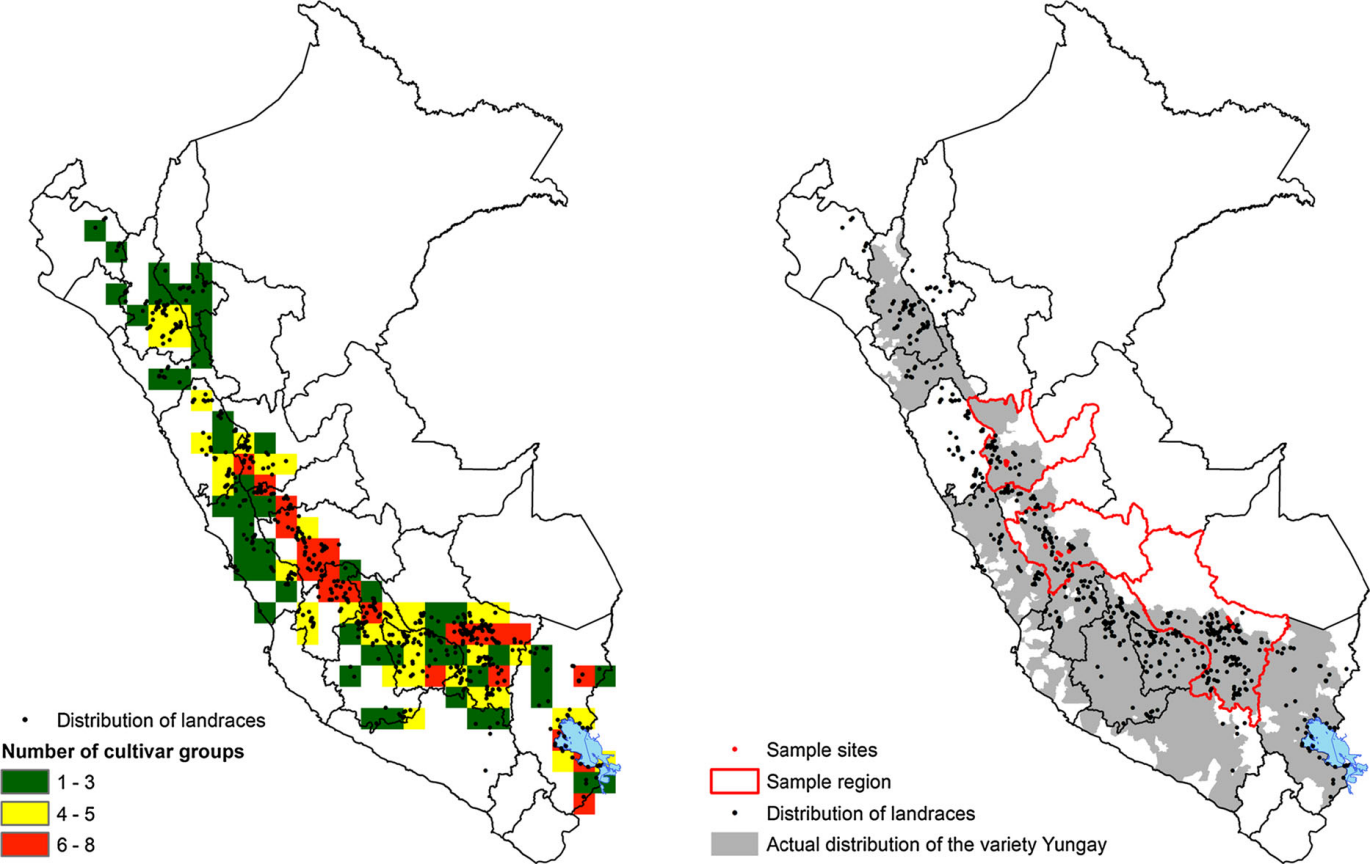
Maps of sampling sites in the Peruvian Andes. *Left* number of cultivar groups of landraces species per 50 × 50 km grid cell as color code for landrace biodiversity. *Right* regions for sampling sites were selected where the improved variety ‘Yungay’ (*grey area*) is cultivated close to landraces (*black dots*). Sampling sites (*red dots*) were located north to south in Junín (n = 244), Huanuco (n = 1,284), and Cusco (n = 143)

On the basis of data available at CIP’s genebank on areas of potential cultivation of landraces and commercial potatoes, we developed and overlaid several maps (Fig. 1). Sympatric condition is observed for a large proportion of the potato area at a resolution of 50 km. However, in the rest of the overlapping area, additional criteria need to be met. These include fertility of the commercial potato variety, coincidence of flowering periods, abundance of pollinator insects, physical distance within the range of insect movement space, seed viability, germination from berries, hybrid fitness, and adoption by farmers.

### Commercial potato variety choice

We used existing data on commercial potato production in the Andes and interviewed potato experts to identify sampling sites in places where coexistence was expected to have occurred over a long period of time. Farmers, chosen randomly, were surveyed and 39 answered several questions corresponding to our selection criteria (Table 1). Thirty-seven farmers reported ‘Yungay’ as one of their commercial varieties that have been cultivated 7–30 years, with an average of 20 years. The survey confirmed the relevance of the potato experts’ advice and therefore validates the purposive, non-probabilistic sampling method used in the present study.

**Table 1:** Characteristics of existing commercial potato varieties currently grown in the Peruvian Andes (from Andean farmer survey)

Variety	Farmer’s variety	Duration (years)^a^	Sympatric with landraces^a^	Fertility
Yungay	37	19 years on average	Very frequent, on average with 3–12	Male and female
Canchán INIAA	14			Male (low %) and female
Perricholi	11			Female
Mariva	3			Male and female
Amarillis	1			Unknown
Capiro	1			Male and female
Tomasa Condemayta	1			Male and female
Unica	1			Male and female

^a^Survey for ‘Yungay’ only

**Fig. 2:**
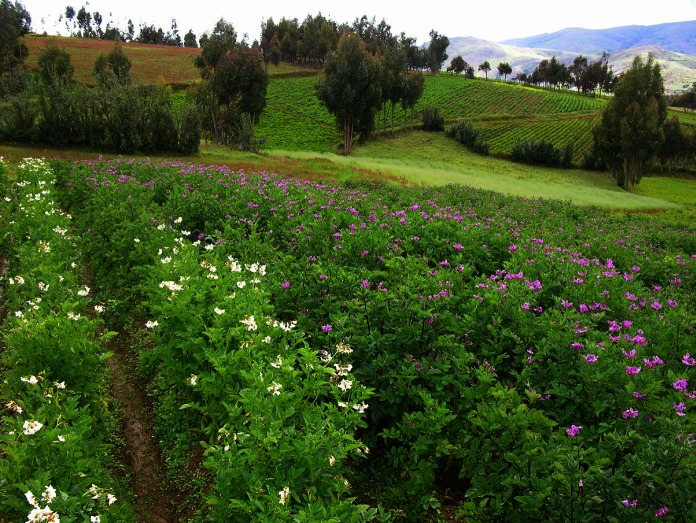
Coexistence of landraces and commercial potato in farmers’ fields in the Peruvian Andes (La Libertad). A landrace producing a yellow-fleshed tuber, ‘Papa Amarilla’ (*white flower on left*), is grown alongside the variety ‘Yungay’ (*purple flowers on right*)

Accordingly, ‘Yungay’ was the only commercial variety that met our four selection criteria. It was released almost 30 years ago and is still highly regarded among farmers in the central Andes. It was bred in part from *Chilotanum* germplasm, which makes it exotic when compared to the Andean varieties. It is fertile and sympatric with the landraces due to its resilience in the highlands environment over 3,000 masl, where it was commonly found growing alongside the landraces (Fig. 2).

The regions we selected for sampling were those where ‘Yungay’ and landraces were cultivated close to each other: Junín, Huánuco, and Cusco. It is here that past hybridization events were most likely to have occurred (Fig. 1). Fields were sampled in several provinces and communities within these regions (Table 2). In farmers’ fields, we sampled all landraces grown and, in some cases, also the commercial potato ‘Yungay’ as referred by the farmers themselves.

**Table 2:** Characteristics of sampling sites in three Peruvian regions in the Andes

Regions	Community	Number of fields	Number of varieties
Junín	Ñuñunguayo	6	30
	Masmachicche	5	50
	Chuamba	6	20
	Chicche	7	300
	La Libertad	5	20
	CasaBlanca	7	20
	Aramachay	2	50
	Pomamanta	4	300
Huánuco	Huacora	6	100
	Monte Azul	3	100
	Huamalli	8	150
	Rodeo de Margos	10	200
	San Juan de Tingo	5	300
	Callampas Alto	4	300
Cuzco^a^	Paucartambo		
	Uscamarca		100
	Chacllabamba		

^a^Samples obtained from a previous harvest (see “Materials and methods” section)

### Iterative screening for detection of potential hybrid

A total of 1,771 samples were collected in farmers’ fields. On the basis of experts’ opinions and farmers’ surveys we estimate that this sample represents more than the 400 landraces grown by farmers in these three regions at any given time. DNA extractions were successful for 1,671 of them, which were then screened by SSR genotyping. By analyzing SSR marker allele frequency of ‘Yungay’ in the 688 potato accessions of the potato genotyping database, we determined the allele frequency for each SSR marker locus and ranked them (Table 3). Through an iterative process (1 SSR at a time, starting with the SSR marker with least frequent alleles) we eliminated all samples that did not share at least one allele with those of ‘Yungay’. This process ruled out all of the 1,671 samples as putative hybrid between ‘Yungay’ and an unknown native potato. Of the 30 SSR markers, 12 were enough for us to reject all of them as possible hybrid with ‘Yungay’ (Table 4). Of the 25 samples referred to as ‘Yungay’ by farmers, only 8 truly were, whereas 17 were not the original variety. These were likely landraces resembling ‘Yungay’ without being either the true-to-type variety or a hybrid.

**Table 3:** SSR markers and their allele frequency for the commercial potato variety ‘Yungay’ used to genotype the landrace samples

SSR allele	Allele frequency	Locus frequency^a^	SSR allele	Allele frequency	Locus frequency^a^
STG0001.146	0.398	0.532	STI0033.131	0.686	0.695
STG0001.150	0.368		STI0033.149	0.061	
STG0001.157	0.022		STI0033.152	0.038	
STG0010.182	0.432	0.447	STM0031.203	0.388	0.388
STG0010.184	0.037		STM0037.91	0.419	0.457
STG0016.143	0.251		STM0037.93	0.108	
STG0016.151	0.138	0.441	STM0037.97	0.028	
STG0016.154	0.366		STM1052.226	0.268	0.374
STG0025.215	0.642	0.642	STM1052.227	0.059	
STI0001.197	0.340	0.381	STM1052.235	0.157	
STI0001.206	0.023		STM1053.186	0.293	0.443
STI0001.212	0.102		STM1053.189	0.342	
STI0003.158	0.349	0.379	STM1053.190	0.330	
STI0003.167	0.107		STM1064.206	0.625	0.625
STI0003.176	0.025		STM1064.209	0.146	
STI0004.95	0.528	0.538	STM1104.182	0.028	0.091
STI0004.112	0.085		STM1104.190	0.063	
STI0012.186	0.109	0.364	STM1106.157	0.184	0.308
STI0012.189	0.300		STM1106.160	0.044	
STI0012.201	0.038		STM1106.169	0.308	
STI0014.139	0.196	0.456	STM5114.305	0.143	0.48
STI0014.145	0.353		STM5114.308	0.384	
STI0023.193	0.092	0.36	STM5114.314	0.367	
STI0023.214	0.351		STM5121.303	0.333	0.335
STI0030.106	0.266	0.414	STM5121.305	0.004	
STI0030.107	0.030		STM5127.254	0.041	0.15
STI0030.109	0.256		STM5127.266	0.067	
STI0032.136	0.265	0.281	STM5127.288	0.144	
STI0032.145	0.048		STPoAc58.248	0.101	0.411
			STPoAc58.249	0.411	

^a^Based on CIP’s potato genotyping database of 688 accessions

**Table 4:** Screening by iteration of 1,671 samples of landraces for potential hybrids which would share at least one allele with ‘Yungay’

Region	No. of samples	No. of samples sharing one allele with ‘Yungay’											
		STM 1104	STI 0032	STM 1106	STM 5121	STI 0012	STM 0031	STM 1052	STPo Ac58	STG 0016	STM 1053	STM 5127	STI 0023
Junín	244	57	18	17	4	2	2	2	2	2	1	0	
Huánuco	1,284	323	181	77	41	29	27	20	16	15	15	14	0
Cuzco	143	46	3	2	0								

## Discussion

Numerous studies have demonstrated that gene flow can occur in potato between commercial potato and native potato and even wild species (Andersson and de Vicente 2009; Celis et al. 2004; Scurrah et al. 2008). Greenhouse and field studies, as well as forced and natural hybridizations, have long demonstrated that seeds may be formed under specific conditions. The frequent production of unreduced gametes among diploid potatoes makes crossing with tetraploid feasible. Even hybrids with uneven ploidy (triploid and pentaploid) that are sterile can survive since potato landraces are clonally propagated. The domestication of the potato from wild species, along with its subsequent diversification into several cultivar groups of different ploidy including hybridization with other wild species, is proof of an active gene-flow process. Evolution of the cultivated potato has been revised several times accordingly (Hawkes 1990; Ochoa 1990; Spooner and Hetterscheid 2006; Spooner et al. 2007).

The impact of gene flow from exotic and transgenic potato varieties is particularly important in the center of origin and diversity from a conservation perspective of the crop’s natural genetic resources. Notwithstanding gene flow of transgene with fitness, which would be relatively easy to monitor, the unintentional introgression of transgene in potato biodiverse germplasm has long been a concern.

Potato gene-flow studies have been conducted in various environments, including the Peruvian Andes. The studies, however, have concentrated almost exclusively on the introgression of transgene into biodiverse potato germplasm to the single gene-flow process. It is actually only the first step of a sequence that may fail at any subsequent step. We recognize that there are at least seven steps involved: the commercial potato must be fertile, the flowering periods must coincide, there must be abundance of pollinator insects, the physical distance must be within the range of insect movement space, seeds must germinate from berries, the resulting hybrid must have fitness, and finally it must be adopted by farmers. Although the latest study by Scurrah et al. (2008) has addressed some of these, there is no clear conclusion as to the probability of success from one step to another. The authors thus advocated conservative measures on restriction of GM potato planting.

Rather than testing each step individually in every environment for every commercial potato variety, we tested ex-post if such unintentional gene flow occurred from commercial potato variety to native potato under conditions where fertility, flowering, pollinator, and sympatry criteria were met. Our study did not detect such gene flow after screening 1,671 DNA samples from more than 400 native varieties grown near a long-standing commercial fertile potato variety in the Peruvian Andes. None of these landraces are hybrids derived from a successful gene-flow event between ‘Yungay’ and a native potato. Hence, we conclude that even when all conditions for gene flow are met, farmers have not unintentionally incorporated genes from the commercial variety ‘Yungay’ into their native potato germplasm.

The immediate question is, what are the reasons for this absence of introgression although hybridization conditions were apparently met? There are a number of unfavorable circumstances around commercial potato: flowers have no nectar and frequently drop off after pollination; pollen is often only partially fertile and spreads to nearby flowers only a few meters away; or insecticides are commonly used in commercial farming, thereby reducing populations of pollinator insects (Andersson and de Vicente 2009). In addition, commercial potatoes, unlike landraces, do not naturalize and are rapidly eliminated by biotic and abiotic constraints (Love 1994; Crawley et al. 2001; Kim et al. 2010). Overlapping flowering has been well-established by previous gene-flow studies (Scurrah et al. 2008). Pollination occurs mainly by bumblebees within relatively short distances generally regarded as being non-significant beyond 10 m (Andersson and de Vicente 2009). Although seed germination from berries and hybrid survival are certainly rare events, we know from the huge native potato diversity that they do occur. When 420 berries with 50–200 seeds each were planted under natural conditions, however, none of the seedlings survived the abiotic and biotic stresses after just two seasons (Scurrah et al. 2008).

We therefore hypothesize that farmers play a predominant role in the absence of a past event of gene flow from the commercial variety ‘Yungay’ to landrace germplasm. This notion has not been recognized enough as a key criterion (if not the ultimate one) for adoption of new native potato arising from hybridization. In a recent publication (de Haan et al. 2010), we highlighted the maintenance of genetic diversity by Andean farmers. One could speculate that farmers would be more inclined to adopt hybrids among landraces rather than between landraces and commercial potato. This is supported by a farmer preference study showing that 76 % of the preferred hybrids were selfed landraces (Scurrah et al. 2008). Another reason may well be the increasing influence of market forces, which require the maintenance of genetic identity of both commercial varieties and landraces. This is probably particularly true for farmers producing both types of potatoes.

Our ex-post analysis of gene flow from a commercial potato variety to native potato landraces in the potato’s center of origin and diversity illustrates the extremely low frequency of such event when the donor parent does not confer fitness or specific characteristics of interest to farmers. If the GM trait is observed by farmers as useful, they are likely to retain it and eventually maintain it as a new landrace. In light of such results, the environmental risk assessment of the cultivation of GM potato in its center of origin and diversity should focus primarily on the transgene fitness only when sympatric conditions may be met. It must also consider the taxonomic distance between the GM potato and landraces as a criterion for likelihood of adoption by farmers. Such higher degree of resolution of the environmental risk assessment will offer access to the benefits of modern technologies so that Andean farmers can realize necessary productivity gains without a risk to native germplasm.
